# Paediatric Investigators Collaborative Network on Infections in Canada (PICNIC) study of aseptic meningitis

**DOI:** 10.1186/1471-2334-6-68

**Published:** 2006-04-10

**Authors:** Bonita E Lee, Rupesh Chawla, Joanne M Langley, Sarah E Forgie, Mohammed Al-Hosni, Krista Baerg, Entesar Husain, James Strong, Joan L Robinson, Upton Allen, Barbara J Law, Simon Dobson, H Dele Davies

**Affiliations:** 1 Department of Pediatrics, University of Alberta, Edmonton, Alberta, Canada; 2 Department of Pediatrics, University of Calgary, Calgary, Alberta, Canada; 3 Department of Pediatrics, Dalhousie University, Halifax, Nova Scotia, Canada; 4 Department of Pediatrics, University of Manitoba, Winnipeg, Manitoba, Canada; 5 Department of Pediatrics, McGill University, Montreal, Quebec, Canada; 6 Department of Pediatrics, University of Saskatchewan, Saskatoon, Saskatchewan, Canada; 7 Department of Pediatrics, University of British Columbia, Vancouver, British Columbia, Canada; 8 Department of Pediatrics, University of Toronto, Toronto, Ontario, Canada

## Abstract

**Background:**

The seasonality, clinical and radiographic features and outcome of aseptic meningitis have been described for regional outbreaks but data from a wider geographic area is necessary to delineate the epidemiology of this condition.

**Methods:**

A retrospective chart review was completed of children presenting with aseptic meningitis to eight Canadian pediatric hospitals over a two-year period.

**Results:**

There were 233 cases of proven enteroviral (EV) meningitis, 495 cases of clinical aseptic meningitis and 74 cases of possible aseptic meningitis with most cases occurring July to October. Headache, vomiting, meningismus and photophobia were more common in children ≥ 5 years of age, while rash, diarrhea and cough were more common in children < 5 years of age. Pleocytosis was absent in 22.3% of children < 30 days of age with proven EV meningitis. Enterovirus was isolated in cerebrospinal fluid (CSF) from 154 of 389 patients (39.6%) who had viral culture performed, and a nucleic acid amplification test for enterovirus was positive in CSF from 81 of 149 patients (54.3%). Imaging of the head by computerized tomography or magnetic resonance imaging was completed in 96 cases (19.7%) and 24 had abnormal findings that were possibly related to meningitis while none had changes that were definitely related to meningitis. There was minimal morbidity and there were no deaths.

**Conclusion:**

The clinical presentation of aseptic meningitis varies with the age of the child. Absence of CSF pleocytosis is common in infants < 30 days of age. Enterovirus is the predominant isolate, but no etiologic agent is identified in the majority of cases of aseptic meningitis in Canadian children.

## Background

Aseptic meningitis is inflammation of the meninges with sterile bacterial cultures of cerebrospinal fluid (CSF) [1]. Although the most common cause in North America is viral meningitis, the differential diagnosis includes "partially-treated" bacterial meningitis, tuberculous or fungal meningitis, inflammation from a para-meningeal bacterial infection, collagen vascular diseases, and drug-induced meningeal inflammation. Non-polio enteroviruses account for 80 to 90% of cases of viral meningitis where a cause can be determined [2,3].

The use of nucleic acid amplification test (NAT) for enterovirus has allowed for rapid diagnosis of viral meningitis, which may decrease the use of empiric antibiotics and the length of hospital stay (LOS) [2,4,5]. The purpose of this study was to establish the seasonality, clinical and radiographic features, management and short term-outcomes of Canadian children presenting over a wide geographic area with aseptic meningitis of presumed viral origin in the modern era.

## Methods

The study was conducted at eight university-affiliated pediatric hospitals in major urban sites across Canada. Local ethics review boards at all sites approved the study. Health records were searched for all children ≤ 18 years of age discharged 1 January 1998 to 31 December 1999 with one or more of the following International Classification of Diseases – Clinical Modification codes: 1) bacterial meningitis 320.9 2) meningitis 322.9 3) viral meningitis 047.9 4) aseptic meningitis 047.9 or 5) EV meningitis 047.9. The inclusion criteria were 1) an abnormal CSF white blood cell (WBC) count defined as: WBC (×10^6^/L) >35 for age < 30 days; >25 for age 30 to 60 days; >5 for age ≥ 61 days [5], 2) a non-bacterial organism identified in CSF by culture, antigen detection or NAT, or 3) a clinical diagnosis of presumed viral meningitis by the attending physician. The exclusion criteria included 1) isolation of a bacterial pathogen from CSF or blood culture or radiological or histological evidence of bacterial meningitis, 2) fungal or parasitic meningitis, 3) meningitis due to neoplasm, and 4) herpes simplex virus meningitis.

Data was collected on the month of presentation, age and gender of the child, presence of underlying medical conditions, body temperature and symptoms at presentation (headache, nausea, vomiting, meningismus, photophobia, rash, diarrhea, and/or cough), and the results of virologic investigations from the local laboratory and computerized tomography (CT) or magnetic resonance imaging (MRI) of the brain, when completed. The use of acyclovir, antibiotics and corticosteroids was recorded. For inpatients, the LOS was recorded in hours and expressed as portion of a day if less than 24 hours and by number of days if greater than 24 hours. Complications such as seizures, hearing loss, subdural collections, and intensive care unit admission were recorded.

Cases were then classified according to predefined criteria as:

1. Proven enteroviral (EV) meningitis:

a. cases with positive EV NAT or EV culture from CSF, or

b. cases with positive EV culture from throat swabs or stool samples and abnormal CSF WBC count

2. Meningitis with identification of viruses other than EV in CSF

3. Clinical aseptic meningitis – cases with abnormal CSF WBC count defined as above and either no viral investigations or negative viral investigations on CSF, throat swab and stool samples

4. Possible aseptic meningitis – patients designated as having aseptic meningitis by discharge diagnosis, but with normal CSF WBC count or no lumbar puncture (LP) performed

Where RBC contamination was suspected, an adjustment was made by subtracting a factor of 1 per 1000 RBC before the criteria of abnormal CSF WBC was applied [6]. Cerebrospinal protein was classified as normal if it was ≤ 0.9 grams/litre (G/L) for infants < 30 days old and ≤ 0.45 G/L for infants ≥ 30 days [7]. CSF glucose was defined as abnormal if it was less than two-thirds of serum glucose when both values were available [6].

Analyses were performed using with SPSS 11.5 for Windows (SPSS Inc., Chicago, IL, USA). Significant differences among categorical variables were identified using Chi square test or Fisher's exact test where applicable. Normality of quantitative variables was evaluated using Shapiro-Wilk and group medians were compared with Kruskal-Wallis test.

## Results

### Demographics

**Figure 1:**
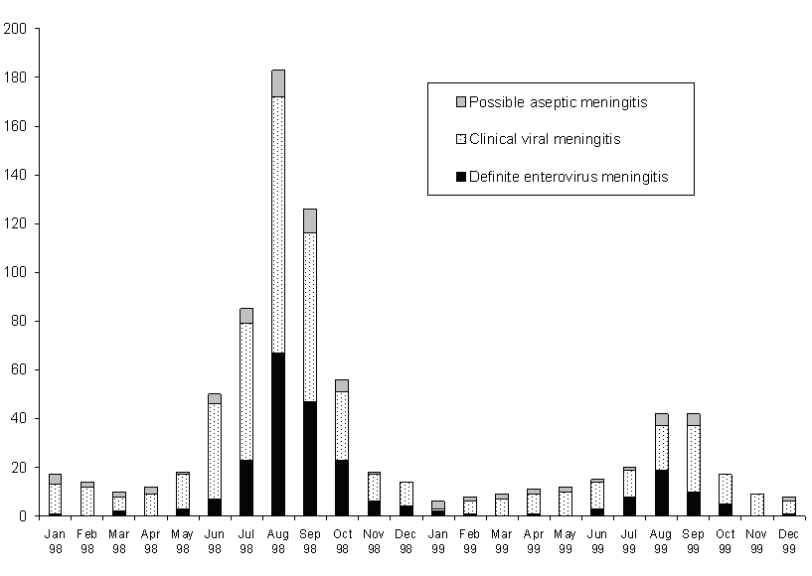
Monthly distribution of proven enteroviral meningitis, clinical aseptic meningitis and possible aseptic meningitis in Canadian children Jan 1998 to Dec 1999.

There were 233 cases of proven EV meningitis, no cases of meningitis with other organisms, 495 cases of clinical aseptic meningitis and 74 cases of possible aseptic meningitis in 802 patients (including 129 outpatients). The seasonal distribution of the cases is shown in Figure 1. Most of the proven EV meningitis and clinical aseptic meningitis occurred from July to October with sporadic cases in other months. An outbreak of aseptic meningitis in the province of Alberta in the summer of 1998 contributed 52% of all cases (Table 1).

**Table 1:** Geographic distribution, age, and viral isolates from 802 children with aseptic meningitis

Institution	No. cases year 1	No. cases year 2	Median age of children year 1 (range)	Median age of children year 2 (range)	Viral isolates in year 1	Viral isolates in year 2
Vancouver, British Columbia†	16	10	7.6 (0.03 – 16.5)	5.6 (0.20 – 15.6)	Coxsackie A09 (N = 1), Enterovirus unspecified type (N = 4)	Enterovirus unspecified type (N = 1)
Calgary, Alberta	179	27	8.2 (0.00 – 18.0)	5.4 (0.10 – 18.0)	Echovirus unspecified type (N = 1), Echovirus 09 (N = 2), Echovirus 30 (N = 25), Enterovirus unspecified type (N = 6), Enterovirus 02 (N = 1), Enterovirus 30 (N = 1), Enterovirus identified by PCR unspecified typed (N = 5)	Echovirus 30 (N = 1)
Edmonton, Alberta	174	37	9.5 (0.01 – 18.0)	7.2 (0.00 – 18.5)	Coxsackie B (N = 1), Echovirus unspecified typed (N = 3), Enterovirus unspecified type (N = 2), Enterovirus identified by PCR unspecified typed (N = 22)	Enterovirus unspecified type (N = 1), Enterovirus identified by PCR unspecified typed (N = 1)
Saskatoon, Saskatchewan	37	5	8 (0.05 – 18.0)	4.1 (0.5 – 16.3)	Echovirus unspecified type (N = 5)	N = 0
Winnipeg, Manitoba	92	21	7.2 (0.02 – 15.9)	4.6 (0.02 – 11.8)	Coxsackie B (N = 1), Echovirus 02 (N = 1), Echovirus 11 (N = 9), Echovirus 30 (N = 35), Enterovirus unspecified type (N = 4), Enterovirus identified by PCR unspecified typed (N = 14)	Coxsackie A09 (N = 5), Enterovirus unspecified type (N = 3)
Toronto, Ontario†	42	40	0.7 (0.02 – 17.2)	1.5 (0.01 – 16.0)	Enterovirus unspecified type (N = 4)*, Enterovirus identified by PCR unspecified typed (N = 10)	Enterovirus unspecified type (N = 6), Enterovirus identified by PCR unspecified typed (N = 7)
Montreal, Quebec	53	50	1.2 (0.03 – 15.2)	6.4 (0.01 – 15.4)	Coxsackie A09 (N = 1), Coxsackie B2 (N = 3), Coxsackie B3 (N = 1), Echovirus 09 (N = 7), Echovirus 11 (N = 1), Echovirus 30 (N = 4), Enterovirus 70/71 (N = 3), Enterovirus 02 (N = 1)	Coxsackie B2 (N = 2), Coxsackie B5 (N = 1), Echovirus 06 (N = 6), Echovirus 09 (N = 2), Echovirus 11 (N = 6), Echovirus 30 (N = 5), Enterovirus unspecified typed (N = 1)
Halifax, Nova Scotia†	10	9	4.3 (0.00 – 15.2)	0.7 (0.02 – 15.6)	Enterovirus unspecified type (N = 5)	Enterovirus unspecified type (N = 3)

* One of the cases was a two month old with normal CSF (9 WBC & 4000 RBC) and enterovirus unspecified type isolated from site other than CSF

† Sites that reviewed inpatients only

Of the 802 patients, 87% (n = 699) had no underlying medical conditions, 9.4% (n = 75) had reactive airway disease, 2.5% (n = 20) had chronic oral or inhaled corticosteroid use, 3.7% (n = 37) had pre-existing neurological disease, 2.7% (n = 22) had psychiatric disease including attention deficit disorder, 1.3% (n = 10) had renal disease, 1.1% (n = 9) had cardiac disease, and < 1% had immunodeficiency (n = 6), malignancy (n = 5), hematologic disease (n = 3), developmental delay (n = 2), diabetes mellitus (n = 1) or congenital adrenal insufficiency (n = 1). The majority of patients were male (60.5%) and the mean age ± SD was 7.3 ± 5.4 years.

### Clinical presentation

**Figure 2:**
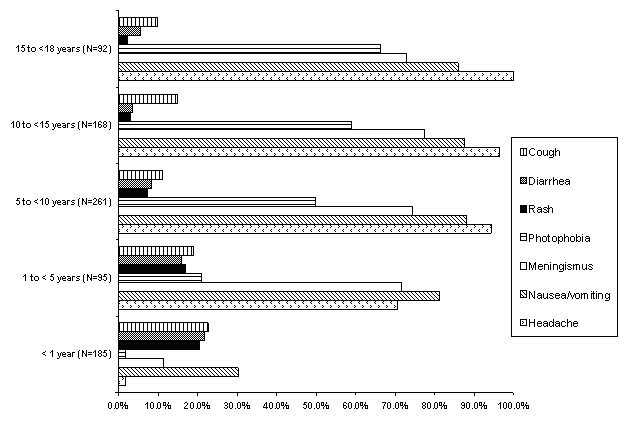
Clinical features of 802 children with aseptic meningitis (One patient was excluded from this analysis as age was not recorded).

Symptoms suggestive of central nervous system infection (headache, nausea, vomiting, meningismus and/or photophobia) were seen more frequently in children 5 years of age and older (99% of older children had these symptoms versus 55% of younger children) (Figure 2) (P < 0.001). However, non-specific symptoms (rash, diarrhea and/or cough) were seen more frequently in children less than 5 years of age (19.3%, 19.6%, 21.4% respectively with 47% having at least one of these symptoms) than in the older children (2.0%, 6.3%, 12.5% respectively with 21% having at least one of these symptoms) (P < 0.001). None of the infants presented with enteroviral sepsis syndrome. The mean temperature at presentation was 38.0 ± 0.9°C with a temperature of ≥ 38.0°C in 409 (54.9%) of the 745 children where it was documented. The mean temperature at presentation for children < 5 years of age was 38.4°C versus 37.8°C for those ≥ 5 years of age (P < 0.001).

### Investigations

At least one lumbar puncture was attempted in 788 of the 802 patients (98.3%). Eight of the 74 cases of possible aseptic meningitis had unsuccessful lumbar puncture, and 14 patients did not have the procedure attempted. Analyzing the 233 cases of proven EV meningitis, 22 patients (9.4%) had normal CSF WBC including 10 of the 31 patients (32.3%) from the < 30-day-old group, 4 of the 19 patients (21.1%) from the 30 to 60 day old group, and 8 of the 182 patients ≥ 61 days old (4.4%) (p < 0.001). Fourteen of the 30 CSF samples (46.7%) from patients < 30 days old and 86 of the 196 CSF samples (44.9%) from patients ≥ 30 days old with proven EV meningitis had abnormal protein (p = 0.8), and most of the cases in all three age groups (where serum glucose was available) had abnormal CSF glucose (148/177, 83.6%). By definition, all 495 cases of clinical aseptic meningitis had abnormal CSF WBC, 234 cases (47.3%) had abnormal CSF protein and 289 of 382 cases with blood glucose results available (75.7%) had abnormal CSF glucose.

*Enteroviridae *was the only family of viruses identified in CSF samples (Table 1). Samples from non-CSF sites identified respiratory viruses including 1 influenza A, 1 adenovirus, 1 parainfluenza virus and 2 respiratory syncytial viruses from 5 patients who fulfilled the criteria of clinical aseptic meningitis with abnormal WBC in their CSF. Viral culture was performed on the CSF of 389 patients (48.5%) and 154 of 389 (38.7%) were positive. The predominant isolate was echovirus 30 (N = 68), with this being most evident in the western sites in the first year of the study. The 2 hospital sites that performed the highest number of viral cultures on CSF had the highest positive recovery rate: 53.4% (55/103) and 53.7% (43/80) respectively. The positive culture rates varied from 5.4% (2/37) to 48.5% (32/66) at the other 6 sites. Nucleic acid amplification test for enterovirus was positive in 81/149 (54.4%) patients with CSF from 19 of these patients positive by viral culture, 35 negative by viral culture and 27 not tested by viral culture. Six patients had positive CSF culture but negative EV NAT (4 enterovirus of unspecific type and 2 echovirus 30). Twenty-seven patients had a positive EV culture from either a throat swab or stool culture. Of these, 6 patients also had positive CSF culture for enterovirus, 3 had positive CSF EV NAT, 9 had negative CSF culture or EV NAT, and 9 had no viral investigations performed on the CSF.

Diagnostic imaging of the head was performed on 158 of 802 (19.7%) of children. Twenty-seven percent (43/158) of these examinations were abnormal (Table 2). Children who had proven EV meningitis were less likely to have diagnostic imaging than were those with clinical aseptic meningitis or possible aseptic meningitis (14.2% versus 20.8% versus 25.7%) respectively, (P < 0.05) or to have abnormal findings other than pre-existing anatomical anomalies or sinusitis (0.0% versus 3.0% versus 6.8%).

**Table 2:** Results of head imaging performed on 158 of 802 children with aseptic meningitis

CT scan result (n = 155*)	MRI result (n = 13*)	Proven enteroviral meningitis (n = 233)	Clinical aseptic meningitis (n = 495)	Possible aseptic meningitis (n = 74)
Normal	Not done	28	70	13
	Normal	1	3	0
	Abnormal findings (extracranial)	0	1^13^	0
Not done	Abnormal findings (intracranial)	1^1^	1^2^	1^3^
Abnormal findings (intracranial)	Not done	0	13^5^	3^6^
	Abnormal findings (intracranial)	0	1^8^	1^7^
Abnormal (previously known findings)	Not done	2^9^	7^10**^	1^11**^
Abnormal findings (extracranial)	Not done	0	4^12^	0
No report	Not done	1	3	0
Number tested/number in diagnostic group (%)		33/233 (14.2%)	103/495 (20.8%)	19/74 (25.7%)
Number abnormal/number in diagnostic group (%)		4/233 (1.7%)	30/495 (6.1%)	5/74 (6.8%)
Number abnormal/number tested (%)		4/33 (12.1%)	30/103 (29.1%)	5/19 (26.3%)

Legend:

CT – computerized tomography

MRI – magnetic resonance imaging

*Ten children had both MRI and CT examinations.

** Includes one child < 30 days of age

^1 ^extra-ventricular obstructive hydrocephalus

^2 ^narrowing of straight sinus

^3 ^inflammatory changes involving thalamus, midbrain and pons

^4 ^one patient had multifocal patchy regions of increased T2, one patient had diffuse cerebral edema and one patient had diffuse increased signal intensity

^5 ^three patients had meningeal enhancement, four patients had a focus of attenuation or hypodensity, three patients had hydrocephalus or ventriculomegaly, one patient had infarct of the cerebellum, basal ganglia, parietal and occipital regions, one patient had optic nerve enlargement, and one patient had a clinical artifact of the brain stem

^6 ^two patients had subdural effusions and one patient had possible increased intracranial pressure

^7 ^focus of attenuation or hypodensity

^8 ^small perforating vessel infarct

^9 ^one patient had calcifications presumably from methotrexate and radiation therapy and one patient had a ventricular peritoneal shunt

^10 ^one patient had old trauma, two patients had Dandy Walker Syndrome, one patient had Cockaynes Syndrome, one patient had a possible small aneurysm, and two patients had post-surgical changes

^11 ^symmetric small ventricles

^12 ^no intracranial abnormalities, but possible sinusitis or mastoiditis

^13 ^no intracranial abnormalities, but possible sinusitis

Acyclovir was administered to 9 patients (3.9%) with proven EV meningitis (median age = 33 days, range: 4 days-16.7 years), 23 patients (4.6%) with clinical aseptic meningitis (median age = 5.7 years, range: 26 days-12.1 years) and 5 patients (6.8%) with possible aseptic meningitis (p = 0.5) (median age = 7.0 years, range: 39 days-17.0 years) (P > 0.05). Antibiotics were used as part of the initial management of meningitis in 594 patients (74.1%) and corticosteroids in 25 patients (3.1%).

A total of 673 patients were admitted to hospital for a median LOS of 2 days. The median length of stay did not vary from 2 days in children with proven enteroviral, clinical aseptic, or possible aseptic meningitis and was not altered by the availability of viral culture or EV NAT. However, LOS ranged from 2 to 4 days at the 8 hospital sites (P < 0.001).

Seventeen of the 802 patients (2.1%) were admitted to the ICU including 2 of the infants < 30 days of age and 5 patients were ventilated. Only one of these patients had proven EV meningitis (a child with a CSF shunt who required ventilation for one day). Possible sequelae were documented for 2 of the 233 patients with proven EV meningitis: a one-month old child had an abnormal evoked potential in the right ear and a 7-year-old child developed aplastic anemia. There were no deaths.

## Discussion

In this large Canadian study of children from 8 urban sites across the country, 802 cases of pediatric aseptic meningitis were identified over a two-year period. Although not population-based, we are confident that our study captured all inpatient cases at the eight pediatric sites from across the country, which account for the majority of tertiary care pediatric beds in Canada.

Meningismus or photophobia was almost universal in older children but only identified in about half of younger children while rash, diarrhea or cough occurred in about one-quarter of older and one-half of younger children. However, lumbar punctures are more frequently performed on young children even in the absence of signs of meningitis and older children with rash, diarrhea, or cough may not have had examination of the CSF. Neonates with proven EV meningitis and no CSF pleocytosis were identified in this study. Similarly, in a previous study where EV NAT was performed on all CSF obtained during EV season, 19% of all cases of proven EV meningitis and 42% of cases in infants < 2 months of age had no CSF pleocytosis [5]. It is not clear if this tendency for infants with EV meningitis to have normal CSF WBC is because of the lower threshold for doing a lumbar puncture in this age group, or because the normal values for CSF WBC used in this and previous studies [5] are too high in this age group. Support for the latter can be found in one study that showed that 11 WBC × 10^6^/L in the CSF is the 90^th ^percentile for infants < 31 days of age [6]. If a threshold of ≥ 20 × 10^6^/L rather than >35 × 10^6^/L had been used to define abnormal CSF WBC in infants < 30 days of age in the current study, 199 (85.4%) patients with proven EV meningitis would have had abnormal CSF, and if a threshold of ≥ 5 × 10^6^/L CSF WBC had been used, 218 (93.6%) of patients would have had abnormal CSF WBC.

Enteroviruses accounted for one-third of the total cases of aseptic meningitis, and about one half of the cases where viral cultures or EV NAT were performed on CSF. Echoviruses accounted for more than two-thirds of all EV isolates with a predominance of echovirus 30 in the first year of the study. This may reflect a greater propensity of this type of enterovirus to invade the central nervous system [3]. Previous North American studies have found that 90% of community-acquired cases of viral meningitis with a proven etiology are due to echoviruses and group B coxsackie viruses [1], with group A coxsackie viruses causing fewer than 5% of cases [9]. Echovirus 13 accounted for only 0.16% of echovirus isolates reported to the US Centers for Disease Control and Prevention from 1970 to 2000, but then accounted for 24% of isolates in 2001, and has now been associated with outbreaks of aseptic meningitis in the United States, Japan, Europe and Israel [8,9]. We did not identify echovirus 13 in CSF in our study, but it is possible that echovirus 13 emerged as a pathogen in subsequent EV epidemics in Canada. Enterovirus 71 has been associated with outbreaks of encephalitis, flaccid paralysis, and aseptic meningitis with a high incidence of neurological sequelae [10] and death due to cardiopulmonary failure [11], and was isolated from non-CSF sites from children in Quebec, Canada during the study period [12]. A possible enterovirus 71 (serotyping could not determine if it was 70 or 71) was isolated from two CSF and one throat swab in the current study. The reason that only *Enteroviridae *were detected in this study and that no etiologic agents were identified in two-thirds of cases is likely that cultures on CSF are not sensitive for other viruses [13]. Furthermore, molecular methods for etiologic agents other than enterovirus, West Nile virus (which had not been described in Canada at the time of this study), herpes simplex virus or *Mycoplasma pneumoniae *have not been validated on CSF and are therefore not routinely applied.

Prospective studies are required to compare patient outcome in the presence or absence of improved viral diagnostics including real-time NAT. Neuroimaging was performed in a minority of patients (most commonly in those who did not have proven EV meningitis) and would not have altered patient management with the possible exception of three patients with hydrocephalus. In a previous study, two of 26 patients (8%) with aseptic meningitis had abnormal CT scans of the brain [5], versus 18 of 115 patients (11.6%) in the current study. Transient CT abnormalities were described in 40% of infants under one year of age with EV meningitis in a study from Japan [14]. These striking differences in the incidence of abnormalities could relate to the age of the patients, the etiology of the aseptic meningitis, the inclusion of patients who actually had meningoencephalitis, and the threshold for imaging the brain.

Almost three-quarters of patients in the current study received antibiotics, as compared with only 23% of patients in an outbreak of EV meningitis in one centre in Germany [15]. Although EV NAT was used in the latter study, positive results were obtained in less than half of patients and were not necessarily available in "real-time", so it is unlikely that availability of NAT accounts for the low use of antibiotics. It is possible that notification of physicians that an EV outbreak is occurring is more efficacious than is use of NAT in individual cases in decreasing the use of antibiotics.

The short-term outcome of Canadian children with aseptic meningitis was generally excellent. A previous study showed that 9% of children less than 2 years of age with aseptic meningitis had coma, complex seizures, or raised intracranial pressure, but these features did not predict long-term neurological sequelae [16]. None of the children in the current study had coma, seizures that were clearly related to meningitis, or proven raised intracranial pressure and long-term sequelae were not assessed. Two studies have shown subtle delays in receptive language in children with EV meningitis in the first three months of life [17,18] with no evidence of other long-term sequelae. No long-term follow-up was performed in the current study.

## Conclusion

In conclusion, EV is the predominant pathogen in Canadian children with aseptic meningitis, but no pathogen is identified in the majority of cases. Absence of pleocytosis is common in young infants with aseptic meningitis. Future efforts at prevention, diagnosis, and therapy of this disease must target EV to have the greatest potential impact, but further studies are required to determine the etiology of non-enteroviral cases.

## Competing interests

The author(s) declare that they have no competing interests.

## Authors' contributions

The study was designed and conducted by RC, JL, and HDD. The data was analyzed by BEL. The manuscript was written by BEL, RC, JL, JLR, and HDD. Data was contributed by BEL, RC, SEF, MA, KB, EH, JS, UA, BL, and SD. All authors read and approved the manuscript.

## Pre-publication history

The pre-publication history for this paper can be accessed here:


